# Synchronous gastric cancer and primary lymphoma of right adrenal gland: a case report

**DOI:** 10.1007/s12328-021-01482-8

**Published:** 2021-07-20

**Authors:** Shuichi Fukuda, Tomoko Wakasa, Hitoshi Hanamoto, Taichi Koyama, Yoshio Ohta, Masatoshi Inoue, Daisuke Terashita, Atsushi Gakuhara, Hideo Tomihara, Katsuya Ohta, Kotaro Kitani, Kazuhiko Hashimoto, Hajime Ishikawa, Jin-ichi Hida, Yutaka Kimura

**Affiliations:** 1 Department of Gastroenterological Surgery, Kindai University Nara Hospital, 1248-1, Otoda-cho, Ikoma, Nara 630-0293 Japan; 2 Department of Pathology, Kindai University Nara Hospital, Nara, Japan; 3 Department of Hematology, Kindai University Nara Hospital, Nara, Japan

**Keywords:** Adrenal lymphoma, Diffuse large B-cell lymphoma, Gastric cancer, Malignant lymphoma

## Abstract

This report presents an extremely rare case of synchronous gastric cancer and primary adrenal diffuse large B-cell lymphoma (DLBCL). An 82-year-old man underwent computed tomography, which revealed a heterogeneous appearing and hypodense adrenal mass and a gastric mass with no enlarged lymph nodes in the neck, mediastinum, abdomen, and inguinal region. Upper gastrointestinal endoscopy revealed a protruding gastric tumor. The specimens obtained from endoscopic biopsy were histologically confirmed to be adenocarcinoma. The hormonal findings eliminated functional adrenal tumor. The patient underwent distal gastrectomy with regional lymph node resection for gastric cancer and incisional biopsy of the adrenal mass. Based on the pathological findings, diagnoses of mixed mucinous and tubular adenocarcinomas of the stomach and adrenal DLBCL were confirmed. Postoperation, the patient received rituximab combined with low-dose doxorubicin, cyclophosphamide, vincristine, and prednisone (R-miniCHOP). Six courses of R-miniCHOP were planned, but were completed in only one course at the patient’s request. The patient died 2 months after surgery.

## Introduction

Gastric cancer is one of the most common gastrointestinal tract carcinomas. Alternatively, primary adrenal lymphoma is extremely rare, accounting for less than 1% of all non-Hodgkin’s lymphomas [1]. Primary adrenal lymphoma is based on a histologically confirmed lymphoma involving unilateral or bilateral adrenal glands without a previous history of lymphoma. Primary adrenal lymphoma requires a significant dominance of adrenal lesions when other organs or lymph nodes are involved by lymphoma concurrently with the adrenal glands [2].

Diffuse large B-cell lymphoma (DLBCL) is an aggressive lymphoma that accounts for about 30% of all cases of non-Hodgkin’s lymphoma [3]. Among all cases of DLBCL, about 40% are extranodal [4]. The synchronous presence of gastric cancer and extranodal DLBCL is rare. In this study, we report an extremely rare case of synchronous gastric cancer and primary adrenal DLBCL.

## Case report

An 82-year-old man was referred to our hospital for further investigation of a right adrenal mass and a gastric mass, which were coincidentally revealed by a follow-up computed tomography (CT) for an abdominal aortic aneurysm. The patient had a history of hypertension, diabetes mellitus, hyperlipidemia, cerebral infarction, and angina pectoris. The patient was an ex-smoker, consuming one pack per year for 55 years, and was a social drinker. In addition, the patient had a 6-kg weight loss in 1 month, abdominal pain, and anorexia. Physical examination showed no swelling of the superficial lymph nodes. The patient’s blood pressure was 139/68 mmHg, pulse was 65 beats per minute, and body temperature was 36.8 °C. The patient had Eastern Cooperative Oncology Group performance status of 1.

**Fig. 1:**
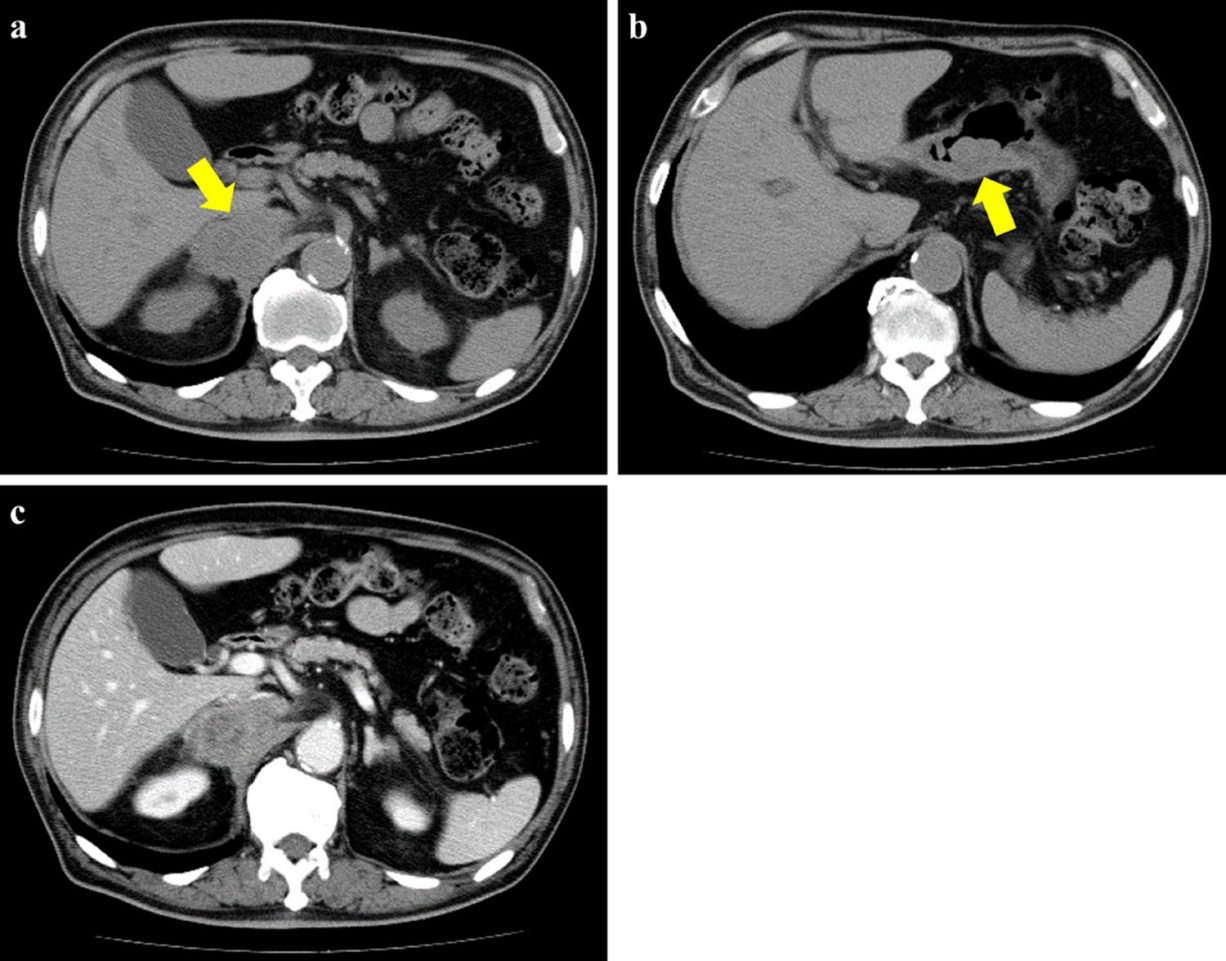
**a, b** Noncontrast-enhanced CT revealed a heterogeneous appearing and hypodense adrenal mass with irregular margins and shape (**a,** arrow), 70 mm in size, and a gastric mass (**b,** arrow), 30 mm in size, with no enlarged lymph nodes. **c** Contrast-enhanced CT revealed a slightly enhanced adrenal mass

**Fig. 2:**
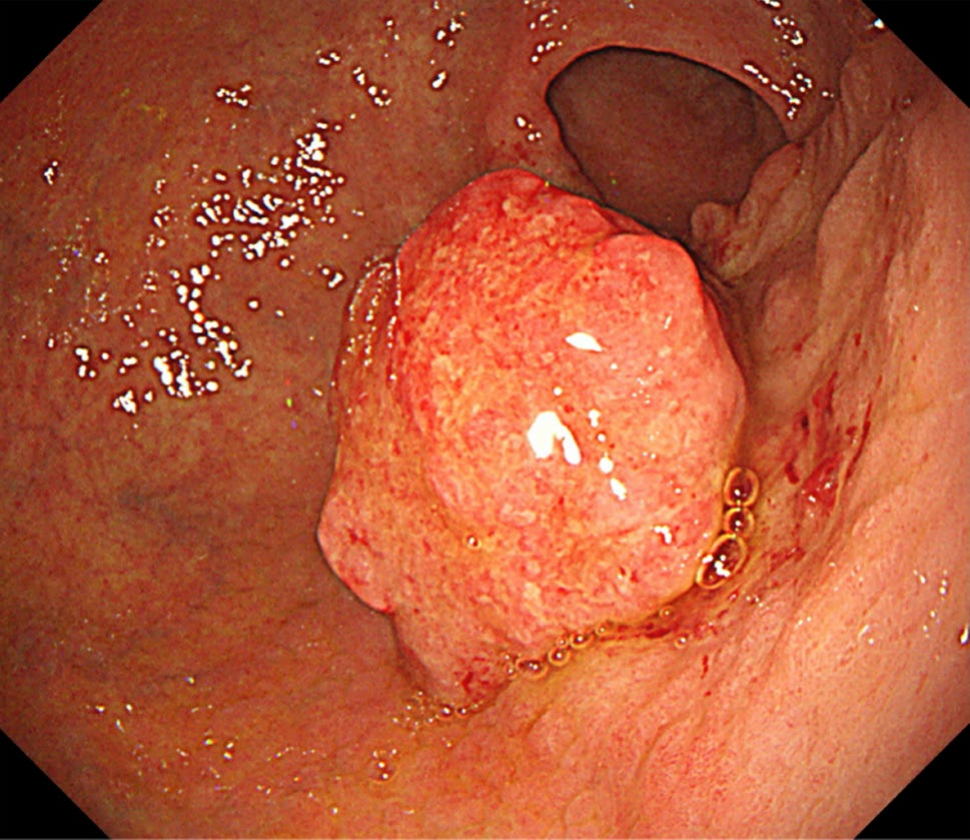
Upper gastrointestinal endoscopy revealed a protruding gastric tumor in the gastric antrum of the posterior wall

Noncontrast-enhanced CT revealed a heterogeneous appearing and hypodense adrenal mass with irregular margins and shape, 70 mm in size, and a gastric mass, 30 mm in size, with no enlarged lymph nodes in the neck, mediastinum, abdomen, and inguinal region (Fig. 1a, b). Contrast-enhanced CT revealed a slightly enhanced adrenal mass (Fig. 1c). Upper gastrointestinal endoscopy revealed a protruding gastric tumor in the gastric antrum of the posterior wall (Fig. 2). The specimens obtained from endoscopic biopsy were histologically confirmed to be adenocarcinoma. Laboratory data, including white blood cell count and sodium level, were within normal ranges. However, lactate dehydrogenase (LDH) level (274 U/L) was elevated in addition to microcytic hypochromic anemia (hemoglobin 9.5 g/dL). Tumor markers, including carcinoembryonic antigen, carbohydrate antigen 19–9, and α-fetoprotein, were within normal ranges. The serum level of soluble interleukin-2 receptor (sIL-2R) was elevated [916 U/mL vs. < 496 U/mL (normal level)]. The plasma levels of adrenocorticotropic hormone, renin, adrenalin, dopamine, and cortisol were within the normal ranges. A decreased plasma level of aldosterone [18.3 pg/mL vs. 35.7–240.0 pg/mL (normal level)] and a decreased urinary level of vanillylmandelic acid [1.01 mg/day vs. 1.5–4.9 mg/day (normal level)] were observed in addition to a slightly elevated plasma level of noradrenaline [0.59 ng/mL vs. < 0.5 ng/mL (normal level)]. These hormonal findings eliminated the diagnosis of functional adrenal tumor. The adrenal mass was large and nonfunctional, it revealed irregular margins and shape, and enhancement on CT and was coexistent with gastric cancer, and the sIL-2R level was elevated; therefore, we considered malignant tumors, including adrenal metastasis from gastric cancer, adrenocortical carcinoma, and adrenal lymphoma. After a discussion at a multidisciplinary cancer conference, gastrectomy, and right adrenalectomy were planned.

Surgical exploration of the abdomen revealed no evidence of ascites or metastasis to the liver or peritoneum. Distal gastrectomy with regional lymph node resection was performed for gastric cancer. The adrenal gland mass was tightly adherent to the surrounding tissues; therefore, the mass was judged to be impossible to resect, and an incisional biopsy was performed.

**Fig. 3:**
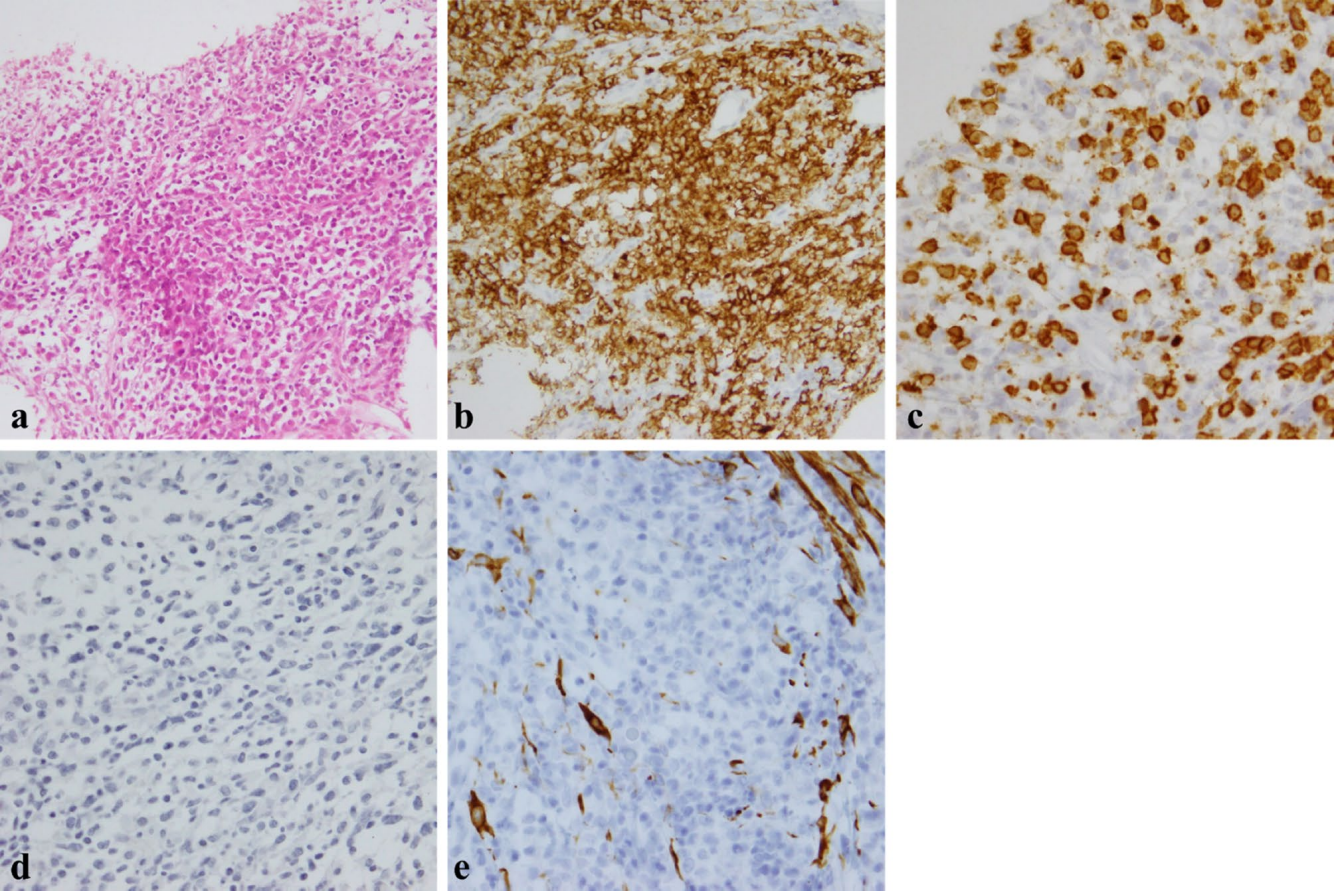
**a** Hematoxylin and eosin staining of the adrenal mass showed a diffuse proliferation of large and atypical lymphocytes accompanied with small lymphocytes. **b**–**e** Immunohistochemical staining revealed that large and atypical lymphocytes were positive for CD20 (**b**) and negative for CD3 (**c**), cytokeratin AE1/AE3 (**d**), and desmin (**e**). The small CD3 + non-lymphoma T cells form the cellular infiltrate around the large and atypical lymphoma B cells (**c**)

**Fig. 4:**
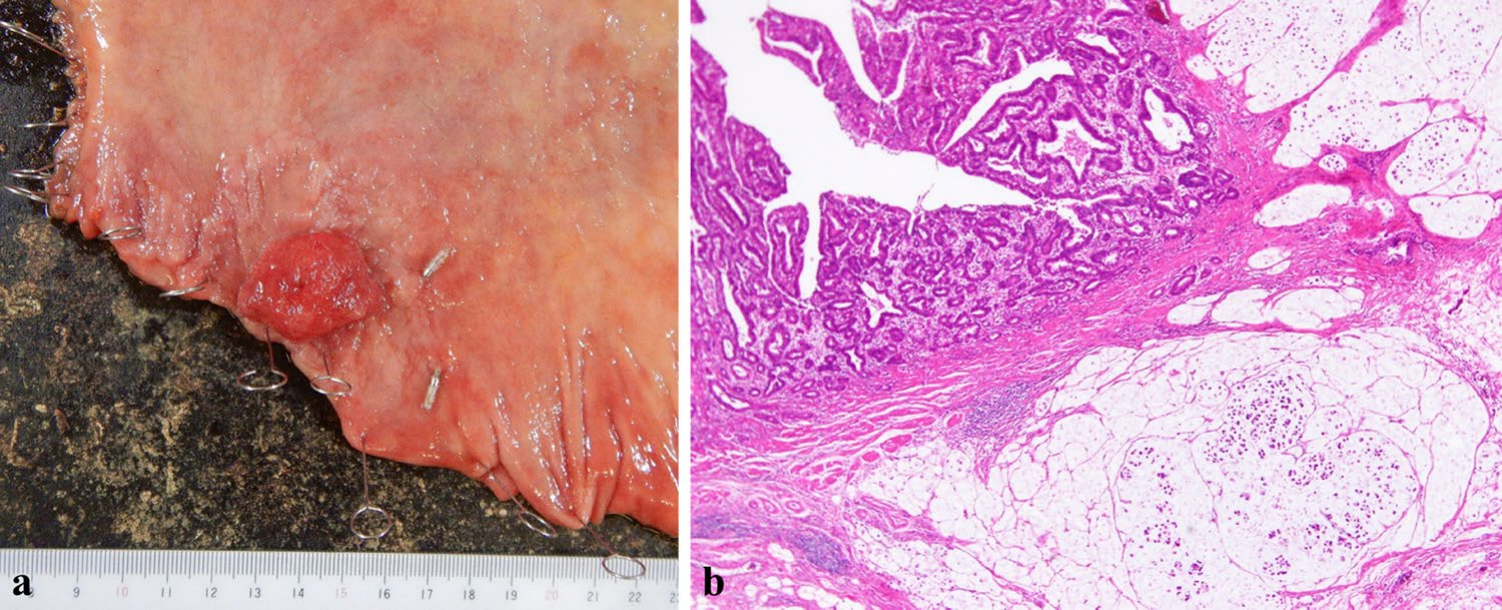
**a** The size of the resected gastric specimen was 50 × 50 mm. **b** Hematoxylin and eosin staining of the gastric mass showed mixed mucinous and tubular adenocarcinomas

Hematoxylin and eosin staining of the adrenal mass showed a diffuse proliferation of large and atypical lymphocytes accompanied by small lymphocytes (Fig. 3a). Immunohistochemical staining revealed that large and atypical lymphocytes were positive for CD20 (Fig. 3b), bcl-2, and leukocyte common antigen and negative for CD3 (Fig. 3c), cytokeratin AE1/AE3 (Fig. 3d), chromogranin A, synaptophysin, desmin (Fig. 3e), myeloperoxidase, CD34, and IgG4. These histopathological and immunohistochemical findings were consistent with DLBCL. The size of the resected gastric specimen was 50 × 50 mm (Fig. 4a). Hematoxylin and eosin staining of the gastric mass revealed mixed mucinous and tubular adenocarcinomas (Fig. 4b). HER2 immunohistochemical staining was negative. No *Helicobacter pylori* were detected by Giemsa staining in the resected specimens. The resection margins were free of tumor cells. The tumor had invaded the submucosal layer at a depth of 4 mm in the submucosa. The resected regional lymph nodes revealed no evidence of metastasis. The patient was diagnosed with T1bN0M0, stage IA according to the 8th edition of the Union for International Cancer Control TNM Classification of Malignant Tumors.

Bone marrow aspiration indicated no bone marrow invasion of DLBCL. The patient had stage IE according to the Ann Arbor staging system. According to the National Comprehensive Cancer Network International Prognostic Index [5], two adverse factors were applicable [age of more than 75 years (3 points) and LDH ratio of more than 1–3 (1 point)], and the risk group was high-intermediate (4 points). The patient received rituximab combined with low-dose doxorubicin, cyclophosphamide, vincristine, and prednisone (R-miniCHOP). The size of the adrenal tumor was unchanged on CT after one course of R-miniCHOP. Six courses of R-miniCHOP were planned but were completed in only one course at the patient’s request. Then, the patient was transferred to a palliative care ward of a hospital and palliative medicine was introduced. The patient died 2 months after surgery.

## Discussion

Gastric cancer is the fourth most common cancer and the second most common cause of cancer-related death worldwide [6]. Alternatively, about 150,000 new cases of DLBCL annually occur worldwide, representing about 30% of all cases of non-Hodgkin’s lymphoma [3]. DLBCLs can occur in any organ, and about 40% of DLBCL cases are extranodal [4]. Synchronous multiple primary cancers are defined as two or more tumors occurring within 6 months of each other [7], and among them, synchronous gastric cancer and primary extranodal DLBCL are rare. To the best of our knowledge, 13 cases of synchronous gastric cancer and primary gastric DLBCL and two cases of synchronous gastric cancer and primary small intestinal DLBCL have been reported in English [8–23]. Particularly, the patient in this case report is the first reported case of synchronous gastric cancer and primary adrenal DLBCL in the English literature.

With the development of diagnostic imaging, the number of adrenal tumors found by chance is increasing [24, 25]. Among adrenal lesions larger than 5 cm in diameter on CT, 28.69% are malignant adrenal tumors, including adrenal lymphoma, accounting for 1.20% of all cases [26]; however, CT findings of primary adrenal lymphoma are not specific. Rashidi et al. have reported that in CT studies of primary adrenal lymphomas, the frequency of homogeneous and heterogeneous appearing masses is similar; most masses are hypodense, whereas some are hyperdense; and most enhancement patterns are “slight to moderate enhancement,” whereas some are “no or poor enhancement [27].” Therefore, a definitive diagnosis is only established by resection or biopsy [27]. In the patient in this case report, CT revealed a “heterogeneous appearing”, “hypodense”, and “slightly enhanced” adrenal mass with a size of 70 mm. Although these CT findings are relatively common in adrenal primary lymphoma, no definitive diagnosis could be made based on imaging studies alone, and an intraoperative biopsy provided a definitive diagnosis. On magnetic resonance imaging (MRI), primary adrenal lymphomas generally show a hypointense pattern on T1-weighted imaging and a hyperintense pattern on T2-weighted imaging [27]. Furthermore, positron emission tomography (PET) is useful in monitoring treatment response and detecting recurrence [27]. Regretfully, both MRI and PET were not performed on the patient in this case report.

The adrenal gland is an organ with abundant blood flow and is prone to hematogenous metastasis from malignant tumors [28]. Adrenal metastasis is not rare in advanced gastric cancer, as there are also reports of adrenal metastasis in early gastric cancer [29, 30]. Therefore, although the preoperative size of gastric cancer was not large in this study, adrenal metastasis from gastric cancer was considered a differential diagnosis of adrenal tumors on imaging. Similar to adrenal lymphoma, metastatic adrenal tumors do not have characteristic CT findings [24, 25]. Gastric cancers with distant metastasis are usually non-candidate for surgery, and chemotherapy is the standard of care for those cases if no tumor bleeding or gastrointestinal obstruction exists; however, for gastric cancers with simultaneous adrenal metastasis, gastrectomy, and adrenalectomy have been reported to provide long-term survival [30]. Therefore, we planned to perform gastrectomy and adrenalectomy for gastric cancer without tumor bleeding or gastrointestinal obstruction combined with a suspicious adrenal metastasis from gastric cancer. The adrenal mass was finally confirmed to be adrenal lymphoma according to pathological findings. This case indicates that when an adrenal mass is simultaneously found with gastric cancer, the adrenal mass should be resected or biopsied to make a definitive diagnosis without assuming that the mass is an adrenal metastasis from gastric cancer.

In the present study, incisional biopsy of the adrenal mass was performed concurrently with gastrectomy for gastric cancer. The adrenal mass was diagnosed as a primary adrenal lymphoma, a disease with poor prognosis, and gastric cancer was pathologically diagnosed as stage IA. Through gastrectomy, chemotherapy for primary adrenal lymphoma had become less tolerable. The preoperative gastric cancer was not large in size and had no enlarged lymph nodes, and there was no tumor bleeding or gastrointestinal obstruction. The gastric cancer was of much less concern than the adrenal mass on imaging. Therefore, in the present case, the histopathological confirmation of the adrenal mass should have been performed first by diagnostic laparoscopy without gastrectomy. In this way, the diagnosis of the adrenal mass could have been made less invasive, and chemotherapy would not have been less tolerable by preserving the stomach. When gastric cancer and an adrenal mass are found simultaneously, the diagnostic approach of the adrenal mass needs to be carefully considered, taking into account the malignancy and progression of each tumor, and when gastric cancer is of much less concern than the adrenal mass as in this case, it is necessary to make a diagnosis of the adrenal mass first before treating the gastric cancer.

Secondary involvement of the adrenal glands by a malignant lymphoma has been reported to occur in as many as 25% of patients with the disease [31]. However, primary adrenal lymphoma is rare, accounting for 3% of all cases of primary extranodal lymphomas [1]. Among primary adrenal lymphomas, the most frequent histology is DLBCL, accounting for 76.3–78.6% of all cases, and common symptoms include B symptoms (fever, night sweats, and weight loss), fatigue, abdominal pain, and anorexia, which are consistent with the patient in this case report [27, 32, 33]. Primary adrenal DLBCL is a disease of the elderly, with a median age at diagnosis of 71 years, and more likely to affect men, with a male-to-female ratio of 2:1, which are also consistent with the patient in this case report [1]. The median survival of patients with primary adrenal DLBCL is poor, being only 14 months [1]. In this study, the primary adrenal lymphoma was right unilateral, and the patient did not show adrenal insufficiency, including hyponatremia and hypotension. This finding agreed with the previous studies that all patients with confirmed adrenal insufficiency had bilateral adrenal lymphomas, and no patients with unilateral adrenal lymphoma showed adrenal insufficiency [32, 34]. Chemotherapy with R-CHOP is the widely recognized treatment strategy for primary adrenal lymphoma [2]. R-miniCHOP was administered to the patient in this case report because the patient was elderly, had just undergone gastrectomy, and had multiple comorbidities [35]. Adrenal involvement is a known risk factor for central nervous system relapse [36, 37]. In this study, the patient was transferred to a different hospital, so whether central nervous system relapse occurred or not is unclear.

Table 1 shows 16 cases of synchronous gastric cancer and extranodal DLBCL previously reported in English, including the patient in this case report [8–19, 22, 23]. Fourteen of the 16 patients (87.5%) were male, with a median age of 71 years (range, 47–82 years). The tumor depth of gastric cancer among the 14 lesions (13 patients) was as follows: mucosa, seven lesions (50.0%); submucosa, two lesions (14.3%); muscularis propria, three lesions (21.4%); tumor penetration of the serosa, one lesion (7.1%); and tumor invasion of adjacent structures, one lesion (7.1%). The median tumor size of DLBCL was 65 mm (range, 25–130 mm). In Table 1, one patient (case 7) was in poor condition and could not be treated, one patient (case 8) was not a candidate for chemotherapy because of comorbidities, one patient (case 12) underwent only chemotherapy because diagnostic laparoscopy showed peritoneal carcinomatosis of gastric cancer, one patient (case 13) rejected chemotherapy for DLBCL, and we did not know whether cases 2 and 5 underwent chemotherapy because it was not mentioned in the paper. The other 10 of the 16 patients (62.5%) underwent tumor resection (operation or endoscopic resection) and chemotherapy. As in case 1, long-term survival of more than 120 months is expected with appropriate treatment.

**Table 1:** Characteristics of synchronous gastric cancer and extranodal diffuse large B-cell lymphoma

Case	Author	Age	Gender	Gastric cancer				DLBCL		Therapy	Outcome
				Tumor location	Tumor size (mm)	Tumor depth	Histology	Tumor location	Tumor size (mm)		
1	Nishino	71	Male	U	NM	NM	A	Stomach	70	Operation and chemotherapy	120 months, alive
2	Ishihama	61	Male	L	NM	m	A	Stomach	NM	Operation	NM
3	Ishihama	77	Male	M	NM	m	A	Stomach	NM	Endoscopic resection and chemotherapy	NM
4	Matsushita	57	Male	L	20	mp	A	Stomach	60	Operation and chemotherapy	12 months, alive
5	Ueo	71	Male	U, L	22, 80	sm, m	E, A	Stomach	NM	Operation	NM
6	Trovato	47	Male	L	3	m	A	Stomach	NM	Operation and chemotherapy	10 months, alive
7	Chong	80	Male	R	NM	NM	A	Stomach	NM	None	30 days, dead
8	Casas	81	Male	L	55	mp	A	Stomach	65	Operation	6 months, alive
9	Weng	51	Male	ML	NM	NM	A	Stomach	NM	Operation and chemotherapy	68 months, dead
10	Fukunaga	71	Male	L	NM	se	A	Stomach	NM	Operation and chemotherapy	18 months, alive
11	Liu	65	Male	M	35	mp	E	Stomach	25	Operation and chemotherapy	6 months, alive
12	Carboni	65	Male	NM	NM	si	A	Stomach	60	Chemotherapy	9 months, alive
13	Ma	81	Female	L	15	m	A	Stomach	NM	Endoscopic resection	18 months, alive
14	Hu	61	Male	L	NM	m	A	Small intestine	NM	Operation and chemotherapy	3 months, alive
15	Chen	49	Female	L	30	m	A	Small intestine	130	Operation and chemotherapy	13 months, alive
16	Our case	82	Male	L	50	sm	A	Adrenal gland	70	Operation and chemotherapy	2 months, dead

*DLBCL* diffuse large B-cell lymphoma, *U* upper third of the stomach, *M* middle third of the stomach, *L* lower third of the stomach, *R* remnant stomach, *NM* not mentioned, *m* mucosa, *sm* submucosa, *mp* muscularis propria, *se* tumor penetration of the serosa, *si* tumor invasion of adjacent structures, *A* Adenocarcinoma, *E* Epstein–Barr virus-associated gastric carcinoma

As this study was a single-patient case report, the accumulation of prospective evidence from more patients in multiple institutions should confirm the findings of this case report. Primary adrenal DLBCL are relatively rare; therefore, the number of patients treated in a single institution is limited. However, the current findings provide important information that can contribute to the development of a treatment strategy for the concomitant occurrence of gastric cancer and adrenal mass.

In conclusion, this case report detailed the case of a patient with synchronous gastric cancer and primary lymphoma of right adrenal gland. This case indicates that in patients with concomitant gastric cancer and an adrenal mass, clinicians should be careful not to assume that the adrenal mass is an adrenal metastasis from gastric cancer without histopathological confirmation and need to consider the possibility of adrenal lymphoma. Furthermore, clinicians need to carefully consider the diagnostic approach of the adrenal mass, taking into consideration the malignancy and progression of each tumor.
